# Insights into the molecular mechanisms underlying responses of apple trees to abiotic stresses

**DOI:** 10.1093/hr/uhad144

**Published:** 2023-07-27

**Authors:** Xuewei Li, Ziqing Ma, Yi Song, Wenyun Shen, Qianyu Yue, Abid Khan, Muhammad Mobeen Tahir, Xiaofei Wang, Mickael Malnoy, Fengwang Ma, Vincent Bus, Shuangxi Zhou, Qingmei Guan

**Affiliations:** State Key Laboratory of Crop Stress Biology for Arid Areas/Shaanxi Key Laboratory of Apple, College of Horticulture, Northwest A&F University, Yangling, Shaanxi 712100, China; State Key Laboratory of Crop Stress Biology for Arid Areas/Shaanxi Key Laboratory of Apple, College of Horticulture, Northwest A&F University, Yangling, Shaanxi 712100, China; State Key Laboratory of Crop Stress Biology for Arid Areas/Shaanxi Key Laboratory of Apple, College of Horticulture, Northwest A&F University, Yangling, Shaanxi 712100, China; State Key Laboratory of Crop Stress Biology for Arid Areas/Shaanxi Key Laboratory of Apple, College of Horticulture, Northwest A&F University, Yangling, Shaanxi 712100, China; State Key Laboratory of Crop Stress Biology for Arid Areas/Shaanxi Key Laboratory of Apple, College of Horticulture, Northwest A&F University, Yangling, Shaanxi 712100, China; Department of Horticulture, The University of Haripur, Haripur 22620, Pakistan; State Key Laboratory of Crop Stress Biology for Arid Areas/Shaanxi Key Laboratory of Apple, College of Horticulture, Northwest A&F University, Yangling, Shaanxi 712100, China; State Key Laboratory of Crop Biology, Shandong Collaborative Innovation Center for Fruit and Vegetable Production with High Quality and Efficiency, College of Horticulture Science and Engineering, Shandong Agricultural University, Tai-An, Shandong 271000, China; Research and Innovation Centre, Fondazione Edmund Mach, San Michele all’Adige 38098, Italy; State Key Laboratory of Crop Stress Biology for Arid Areas/Shaanxi Key Laboratory of Apple, College of Horticulture, Northwest A&F University, Yangling, Shaanxi 712100, China; The New Zealand Institute for Plant and Food Research Limited, Havelock North 4157, New Zealand; Department of Biological Sciences, Macquarie University, North Ryde, NSW 2109, Australia; State Key Laboratory of Crop Stress Biology for Arid Areas/Shaanxi Key Laboratory of Apple, College of Horticulture, Northwest A&F University, Yangling, Shaanxi 712100, China

## Abstract

Apple (*Malus*$ \times $*domestica*) is a popular temperate fruit crop worldwide. However, its growth, productivity, and quality are often adversely affected by abiotic stresses such as drought, extreme temperature, and high salinity. Due to the long juvenile phase and highly heterozygous genome, the conventional breeding approaches for stress-tolerant cultivars are time-consuming and resource-intensive. These issues may be resolved by feasible molecular breeding techniques for apples, such as gene editing and marker-assisted selection. Therefore, it is necessary to acquire a more comprehensive comprehension of the molecular mechanisms underpinning apples’ response to abiotic stress. In this review, we summarize the latest research progress in the molecular response of apples to abiotic stressors, including the gene expression regulation, protein modifications, and epigenetic modifications. We also provide updates on new approaches for improving apple abiotic stress tolerance, while discussing current challenges and future perspectives for apple molecular breeding.

## Introduction

Apple (*Malus*$ \times $*domestica*) is a widely produced and economically important fruit worldwide. It is the preferred choice for people to obtain abundant vitamins and dietary fiber in modern society. Although apple trees are extensively grown in the temperate regions, particularly in Asia, North America, Europe, and Oceania, its productivity and quality are regularly threatened by local fluctuating abiotic stresses caused by climate change.

Drought, extreme temperatures, and high salinity are the most important environmental determinants that are limiting apple growth worldwide, particularly under the current climate change scenarios [1]. Climate change has led to an increased year-to-year and seasonal variability in precipitation, resulting in longer periods of reduced soil water availability for plants [2, 3]. Currently, agriculture uses more than 70% of all freshwater consumed, and the consumption is rising as the environment gets drier. In addition, extreme temperatures in important apple-growing regions, particularly in the Mediterranean and subtropical environments, are having a negative impact on apple production [2, 4, 5]. Soil salinity is another common factor affecting crop production, and it is anticipated that saline soils will become more prevalent in approximately 50% of irrigated lands [6]. Both vegetative and reproductive growth of apple have been reported to be negatively affected by not only the frequency of drought events, extreme temperatures and salt stress, but also their duration and intensity [7, 8]. Therefore, understanding the physiological and molecular mechanisms behind apple responses to abiotic stresses is crucial for sustainable apple production and the development of new cultivars resistant to such challenges.

To cope with abiotic stresses, two main strategies are commonly employed for sustainable apple production: improving agricultural practices and breeding stress-tolerant apple varieties. Traditional breeding methods for stress-tolerant apple cultivars are time- and labor-intensive because of the self-incompatibility and long juvenile period of apples. Fortunately, biotechnological approaches such as genetic transformation and genome editing have demonstrated the potential to enhance the stress tolerance of apple [9–11]. Thus, unraveling the molecular mechanisms underlying the responses of apple trees to abiotic stresses is critical for breeding of abiotic stress-tolerant cultivars and enhancing apple yield and quality.

Currently, there is a strong focus on understanding how apple trees respond to abiotic stresses and how they perceive and transmit stress signals to downstream components during stress responses. In this review, we aim to summarize the recent progress made in the study of molecular regulation at various levels, including transcriptional, post-transcriptional, post-translational, and epigenetic levels. It is noteworthy that the aforementioned findings hold great significance for the development of stress-resistant apple varieties and enhancing both yield and quality of apples.

### Transcriptional regulation

**Figure 1:**
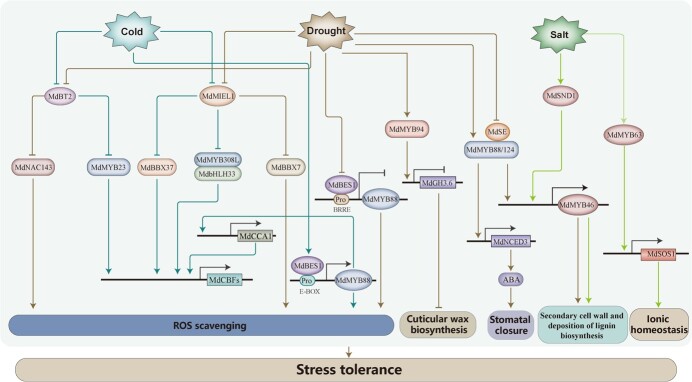
Overview of the representative transcriptional regulation mediated by transcription factors in apple in response to cold, drought, and salt stresses.

Abiotic stresses induce profound changes in the apple transcriptome. It has been estimated that approximately 25% of the apple genome is comprised of cold-regulated genes, while drought-regulated genes make up around 10% of the genome [12, 13]. Gene expression mediated by transcription factors and other functional genes constitutes a fine-tuned transcriptional network in apple. Transcription factors (TF) act as molecular switches that regulate the plant stress responses by binding to the *cis*-elements of downstream gene promoters and mediating their expression [14–16]. Apart from the DNA-binding domains, which bind to the promoters of down-stream genes, most of the transcription factors also contain nuclear localization signals and specific motifs [17]. A number of transcription factor families play important transcriptional regulatory roles in abiotic stress responses of apple, including NAM, ATAF1/2, and CUC2 (NAC) [18, 19]; v-myb avian myeloblastosis viral oncogene homolog (MYB) [20–22]; zinc finger protein (ZFP) [23, 24]; basic helix–loop–helix protein (bHLH) [25–27]; proteins with WRKYGQK heptapeptide at the N-terminal end (WRKY) [28, 29]; ethylene responsive factor (ERF) [26, 30, 31]; heat shock factor (HSF) [32, 33], and basic leucine zipper (bZIP) transcription factors [34–36]. These transcription factor families share the common feature that the majority of their members respond rapidly to abiotic stress conditions (Fig. 1).

#### AP2/ERF regulon

Drought generally refers to an imbalance in water supply and demand during the apple tree’s growth cycle or a continuous water shortage as a consequence of a low water budget, which results in leaf wilting, yellowing, and advanced abscission, as well as premature fruit ripening and drop [37]. In apple trees, drought stress causes a decrease in leaf area and temporary cessation of stem growth due to impaired plant carbon balance caused by stomata closure and hydraulic failure [38]. Drought stress during hot summers can often result in leaf scorching due to the accompanying heat stress. As a result of frequent drought stress events caused by dry weather resulting from global warming, the apple yield and quality have significantly decreased [39]. Therefore, it is important for apple trees to sense soil water availability and initiate appropriate molecular stress responses to survive during such drought stress conditions. To date, numerous genes in the APETALA2/ethylene responsive factor (AP2/ERF), ZFP, bHLH, MYB, and NAC families have been identified and characterized to take part in apple drought responses (Table 1).

**Table 1:** Transcription factors involved in apple drought stress response

Mechanisms	AP2/ERF	AP2/EREBP	ZFP	bHLH	MYB	NAC	WRKY
Stomatal closure	MsDREB6.2 [40]			MdbHLH130 [27]	MdMYB88/124 [41–43]		
				MdbHLHm1 [25]			
ROS scavenging	MsDREB6.2 [40]		MdBBX10 [44]		MdMYB94 [45]	MdNAC1 [18]	MbWRKY5 [46]
			MdDof54 [47]			MdNAC143 [48]	MbWRKY1 [49] MbWRKY3 [50]
			MdBBX7 [23]				MbWRKY2 [51]
Cuticular wax biosynthesis		MdSHINE2 [52]			MdMYB94 [45]		
Anthocyanin biosynthesis	MdERF38 [30]				MdSIMYB1 [53] MdMYB1 [30]		
Root development	MsDREB6.2 [40]		MdBBX10 [44]		MdMYB88/124 [21]		
	MpDREB2A [54]		MdBBX7 [23]		MdSIMYB1 [53]		
	MhDREB2A [55]		MdDof54 [47]		MdMYB94 [45]		
			MdZAT10 [55]				
miRNA biogenesis			MdZAT5 [56]				
Chlorophyll stability							MdWRKY17 [57]

*DREB* (dehydration-responsive element-binding) genes encode AP2/ERF family proteins and usually function as transcription factors by interacting with ethylene-responsive sequence motifs, including GCC-box and DRE/CRT (dehydration responsive element/C-repeat) motifs. When facing drought stress, the *DREB*s are rapidly induced, and their accumulation usually positively regulates the drought stress tolerance of apple trees. For example, ectopic expression of *MsDREB2C* and *MsDREB6.2* isolated from *Malus sieversii* [40, 58], *MpDREB2A* from *Malus prunifolia* [54], *MbDREB1* from *Malus baccata* [59]*,* and *MdDREB76* from *Malus domestica* [60] all confer the drought tolerance in transgenic Arabidopsis or tobacco. In apple, overexpression of *MsDREB6.2* also improves the ability of transgenic plants to withstand drought conditions by binding to the promoter of cytokinin (CTK) biosynthetic gene *MdCKX4a* and aquaporin genes, inducing their expression [40]. This results in a reduction in endogenous CTK levels of roots, causing a more developed root system and a lower shoot/root ratio in *MsDREB6.2*-overexpressed apple plants. The elevated aquaporin genes induced by MsDREB6.2, including *MdPIP1;3* and *Mdγ-TIP* promote the root hydraulic conductance in these plants [40]. The *MsDREB6.2* homolog from *M. domestica*, *MdDREB2A*, also confers the drought tolerance in apple by facilitating the expression of *MdCKX4a* [61]. A similar biological function responding to drought condition has also been found for MhDREB2A, which originates from the drought tolerant rootstock ‘SH6’ (*Malus honanensis*). MhDREB2A is found to be directly involved in inducing *MhZAT10* expression in response to drought stress. As a transcription factor, MhZAT10 further associates to the promoter of *MhWRKY31*, resulting in the induction of its expression. This confirms that the MhDREB2A-MhZAT10-MhWRKY31 module plays an essential role in the apple plant’s response to drought stress condition [55]. The conserved function of DREB2A suggests its involvement in activating downstream genes during the apple drought stress response (Table 1). Additionally, the MhDREB2A-MhZAT10 module participates in apple cold stress response [55].

During frost conditions, different apple tissues may experience low temperature stress that exceeds their capability of supercooling [62]. For example, while the bark and leaf buds are usually uninjured at −60°C, the xylem is damaged at −35°C to −45°C [63]. Although apple trees can withstand extreme cold temperatures, their production traits, such as fruit weight, size, sweetness, firmness, and yield, are significantly impacted [64]. Therefore, deciphering how apples respond to low temperature stress at a molecular level can assist in the development of cold-hardy apple cultivars.

Numerous studies have been conducted on the transcription regulation participated in apple cold stress response (Table 2). Cold stress induces the expression of many genes with promoters containing DRE/CRT cis-acting elements in *M. baccata*, a species with extreme cold tolerance (around −40°C) [65], including CBFs (C-repeat binding factors, also referred to as dehydration-responsive element-binding protein 1 s or DREB1s). CBFs are well-known transcription factors that play a crucial role in the plant’s response to cold stress. They bind to the DRE/CRT *cis*-acting elements of COR (COLD RESPONSIVE) genes, which leads to the activation of their expression [66, 67]. The apple genome contains five CBF genes, and one of them, MbDREB1, isolated from the crab apple *M. baccata*, confers plant tolerance to low temperature *via* both ABA-dependent and ABA-independent pathways [59]. Another cold responsive gene is MhZAT10, which acts as the downstream TF of MhDREB2A. Furthermore, the yeast one-hybrid assay shows that MhZAT10 binds to the promoters of *MhMYB88* and *MhMYB124*, which are the core positive regulators in apple cold stress response [55, 68]. Hence, MhDREB2A and MhZAT10 serve as converging nodes in the transcriptional networks that regulate apple responses to both drought and cold stress.

**Table 2:** Transcription factors involved in apple cold stress response

Mechanisms	MYB	NAC	ERF	bHLH	ZFP
CBF dependent	MdMYB23 [69]	MdNAC029 [19]	MdERF1B [26]	MdCIbHLH1 [70]	MdBBX37 [71]
	MdMYB23MYB88/124 [68]				
	MdMYB108L [72]				
Anthocyanin biosynthesis	MdMYB23 [69]			MdbHLH33 [73]	MdBBX20 [24]
	MdMYB308L [73]			MdbHLH3 [74]	MdBBX37 [71]
	MdMYB88/124 [23, 68]				
ROS scavenging	MdMYB88/124 [68]	MbNAC25 [75]			MhZAT10 [55]
Ethylene biosynthesis			MdERF1B [26]	MdCIbHLH1 [26]	
			MdERF3 [26]		
JA mediated response					MdBBX37 [71]
Lignin biosynthesis	MdMYB46 [76, 77] MdMYB83 [77]				
Target gene expression	MdMYB46 [76]	MdSND1 [77]			MhZAT10 [55]

MdERF38 is a member of the AP2/ERF family and functions as an ethylene response factor. It has been demonstrated to enhance drought stress tolerance in apple trees by promoting the accumulation of anthocyanins [30]. Acting as an upstream regulator, MdERF38 can bind to the promoter of *MdMYB1*, resulting in anthocyanin production that enhances apple drought tolerance by scavenging reactive oxygen species [30]. In addition, the apple APETALA2/ethylene-responsive element binding proteins (AP2/EREBP) transcription factor MdSHINE2 contributes to apple drought tolerance by facilitating the biosynthesis of leaf and stem cuticular wax, a primary barrier for leaves to regulate non-stomatal water loss [52].

In apple, ERFs play roles in the response to salt stress by regulating the expression of *SOS1* (salt overly sensitive 1). *SOS1* is the most extensively researched gene involved in the response to salt stress. It is a transporter located on the plasma membrane that facilitates Na^+^ efflux from the cytoplasm with the assistance of plasma membrane H^+^-ATPases [78, 79]. Apple MdERF106 has been shown to interact with MdMYB63, enhancing the binding capacity of MdMYB63 to the promoter of *MdSOS1*. This, in turn, positively regulates the salt stress tolerance of apple trees [31]. Meanwhile, MdERF4, another ERF transcription factor, functions as a suppressor of salt stress tolerance in apple. It has been suggested that MdERF4 binds to the DRE motif of the *MdERF3* promoter and inhibits its expression [80] (Table 3). Moreover, crosstalk between an ERF and a NAC transcription factor has been observed when apple is exposed to salt stress. MdNAC047 can bind to the promoter of *MdERF3* to form a module that mediates ethylene biosynthesis and confers salt stress tolerance [81]. These documented molecular events suggest various regulatory roles of apple ERF TFs in governing downstream signaling pathways, which enable apple plants to appropriately respond to drought, cold, and salt stress conditions.

**Table 3:** Regulatory mechanisms of apple transcription factors involved in salt response

Mechanisms	MYB	NAC	ERF	bHLH	WRKY
Regulating *MdSOS1* expression	MdMYB63 [31]		MdERF106 [31]		
ABA response				MdSAT1 [25]	MdWRKY30 [28]
Ethylene biosynthesis		MdNAC047 [81]	MdERF3 [80]		
			MdERF4 [80]		
ROS scavenging		MdSND1 [77]	MdDREB76 [60]	MxbHLH18 [82]	MdWRKY100 [83]
					MxWRKY55 [84] MxWRKY64 [85] MxWRKY53 [86] MbWRKY5 [46] MbWRKY4 [87]

#### ZFP regulon

ZFPs, which contain a zinc-finger domain stabilized by zinc and can interact with DNA, RNA, or proteins [88, 89], are also involved in apple’s response to drought stress. BBX (B-BOX) is a large class of ZFPs containing one or two B-box motifs and sometimes a CCT domain at the C-terminus [90]. In the apple genome, a total of 64 BBX TFs have been identified, most of which are induced by multiple abiotic stresses [90]. Among them, MdBBX10 enhances the ability of plants to scavenge ROS (reactive oxygen species) during stressful conditions, thereby improving the tolerance of transgenic Arabidopsis to drought and salt stress conditions [44]. Additionally, MdBBX7 improves apple trees’ drought tolerance by activating the expression of *MdERF1*, *MdERD15*, and *MdGLK1* [23]. Chromatin Immunoprecipitation (ChIP-seq) analysis has also revealed that MdBBX7 identifies the conserved T/G-box and CCTTG elements present in the promoters of its target genes, including *MdERF1*, *MdERD15*, and *MdGLK1*. These findings indicate that MdBBX7 plays an essential role in regulating the molecular response to drought stress in apple trees, and provides valuable insights into the mechanisms underlying this process. To further elucidate these mechanisms, yeast two-hybrid screening identifies MdMIEL1 (MYB30-interacting E3 Ligase 1) as a protein interacting with MdBBX7. MdMIEL1 encodes a ubiquitin E3 ligase and degrades MdBBX7 through the 26S protease pathway. Genetic analysis has shown that MdMIEL1 has an epistatic effect on MdBBX7, negatively modulating apple drought stress [23]. Furthermore, MdMIEL1 negatively regulates cold stress tolerance in apple by facilitating the ubiquitination and degradation of MdBBX37 [91]. Additionally, MdBBX37 interacts with both MdICE1 and MdJAZ1/MdJAZ2 (JA signaling repressors, JAZMONATE ZIM-DOMAIN), which can inhibit the transcriptional activation of *MdCBF1* and *MdCBF4* by MdBBX37. Interestingly, when working together with MdICE1, this complex synergistically enhances the transcriptional activity of MdICE1 on *MdCBF1*. Therefore, the BBX37-ICE1-CBF module is regulated by both MIEL1 and JAZ to co-regulate the JA-mediated cold stress tolerance in apple [91] (Fig. 1).

ZAT is a type of C2H2 zinc-finger transcription factor which plays a crucial role in plants’ responses to abiotic stress. Apple MdZAT5 is upregulated in response to PEG-induced (polyethylene glycol) drought stress in roots of *M. sieversii*, which is a widely used rootstock of apple under abiotic stress conditions [92]. Interfering with the expression of *MdZAT5* in apple plants leads to a hypersensitive phenotype under drought stress conditions. Conversely, over expression of *MdZAT5* has been shown to enhance many drought-responsive genes expressions in response to drought stress, resulting in increased drought tolerance in transgenic apple plants [56]. Interestingly, ChIP-seq analysis has identified the conserved binding motif of MdZAT5 as T/ACACT/AC/A/G. Moreover, MdZAT5 has been shown to interact with and directly target HYPONASTIC LEAVES1 (MdHYL1), which facilitates the biogenesis of drought-responsive miRNA in response to drought stress [56]. Although *MdZAT5* is upregulated upon drought and ABA treatment, it is suppressed under salt and cold stresses [93]. Overexpression of *MdZAT5* in apple calli and *Arabidopsis* has been found to promote the anthocyanin accumulation and reduce the ability of transgenic Arabidopsis plants and apple calli to tolerate salt [93]. Another C2H2 zinc-finger protein, MhZAT10, functions as a positive modulator of *MhWRKY31*, *MhMYB88*, and *MhMYB124*. It binds to the promoter of *MhWRKY31*, leading to its increased expression and imparting drought tolerance to the plant. Moreover, MhZAT10 has been found to mediate the upregulation of *MhMYB88* and *MhMYB124*, thereby enhancing cold tolerance in apple trees [55]. These findings suggest that MhZAT10 has a crucial function in mediating crosstalk between drought and cold stress responses, highlighting the importance of MdZATs in managing abiotic stress responses in apple trees.

Dof (DNA-binding one zinc-finger) transcription factors, including *MdDof24*, *MdDof6*, *MdDof26*, and *MdDof54* [47, 94, 95], are another type of ZFPs that play pivotal roles in the abiotic stress response of apple. However, among them, only the molecular mechanism of MdDof54 has been reported. MdDof54 recognizes the promoters of its downstream genes containing AAAG motifs to mediate drought-responsive genes expression under drought stress conditions [47]. Additional research is necessary to clarify the roles of other MdDof transcription factors in apple and to explore their potential in improving apple stress tolerance.

#### MYB regulon

There are 229 MYB gene models in apple genome, including two typical 4R-like MYB proteins, five R1R2R3 MYB proteins, and 222 typical R2R3 MYB proteins. These MYB regulons are further subdivided into 45 subgroups based on sequences similarity [96]. Interestingly, the apple genome contains two or more putative orthologs of MYB, as compared to a single protein present in Arabidopsis, indicating the expansion events of the MYB gene family of apple genome. Among these MYB proteins, the R2R3 MYBs have been the most extensively studied. In particular, 18 R2R3 MYBs have been found to be induced by abiotic stress treatment, including *MYB54, MYB67, MYB97, MYB107, MYB146, MYB148, MYB155, MYB185, MYB197, MYB199, MYB206, MYB222, MYB11, MYB22, MYB109, MYB121, MYB133,* and *MYB136* [96]. Over-expression of *MdoMYB121* in tomato and apple enhances plants’ drought, cold and salt stress tolerance [96]. However, the molecular function mechanism of *MdoMYB121* has not been analysed.

MdMYB88 and MdMYB124 are two paralogous genes that exhibit functional redundancy in apple. They have a vital function in responding to cold stress through both CBF-dependent and CBF-independent pathways. By directly binding to the promoters of CBF-dependent gene *MdCCA1* (Circadian Clock Associated 1) and CBF-independent gene *MdCSP3* (Cold Shock Protein 3), MdMYB88 and MdMYB124 activate their expression under cold stress, therefore conferring cold stress tolerance in apple [68]. Besides MdCSP3 and MdCCA1, MdTIC (time for coffee) is another down-stream gene of MdMYB88. Reduced expression of *MdTIC* results in decreased freezing tolerance and unsaturated fatty acid of apple [97]. The upstream TFs of *MdMYB88* have also been identified to construct the regulation network. The nuclear localized MdBES1 (BRI1 Ethylmethane Sulfonate Suppressor1), a vital component of BR (brassinosteroids) signaling, is identified through a yeast one hybrid (Y1H) screen assay. Under control and drought conditions, MdBES1 binds to the E-box (CANNTG) of the *MdMYB88* promoter, leading to the repression of *MdMYB88* expression. However, under cold stress, MdBES1 binds to the BRRE motif (CGTGTG) of the *MdMYB88* promoter and activates its expression [22]. Moreover, MdMYB88 and MdMYB124 promote BR biosynthesis. Therefore, MdBES1 plays multifaceted roles in apple cold and drought stress response by regulating BR biogenesis. MdMYB88 and MdMYB124 appear as an internode to associate various abiotic stress responsive factors. Firstly, they regulate drought responses by facilitating root xylem development *via* direct targeting the promoters of *MdVND6* and *MdMYB46*, which are key regulators for secondary wall-associated cellulose accumulation and xylem vessel differentiation [21]. Furthermore, MdMYB46 can bind to the M46RE and SMRE motifs in the promoters of lignin biosynthesis-related genes and promote the biosynthesis of secondary cell walls, leading to an enhanced osmotic and salt stress tolerance in apple trees [21, 76, 77]. Secondly, MdMYB88 and MdMYB124 also enhance apple’s ability to tolerate drought stress by directly controlling the expression of genes responsible for phenylpropanoid biosynthesis, leading to the accumulation of chlorogenic acid, catechinic acid, quercetin, and the non-enzymatic antioxidants involved in ROS detoxification [42]. Thirdly, MdMYB88 and MdMYB124 associate with promoter regions of the ABA biosynthetic gene *NCED3* (9*-cis*-epoxycarotenoid dioxygenase 3) to induce the ABA accumulation and confer apple drought tolerance [20]. Fourthly, MdMYB88 and MdMYB124 interact with MdSE (SERRATE), a protein involved in miRNA biogenesis. Under drought stress, MdSE inhibits the expression of *MdMYB88* and *MdMYB124*. This inhibition results in a decrease in transcription levels of *MdNCED3* and an increase in ABA accumulation [43] (Fig. 1). In summary, MdMYB88 and MdMYB124’s regulatory network involves multiple pathways and various downstream genes.

Several MYB TFs have been identified to play roles in apple cold response by mediating anthocyanin biogenesis, which confers stress tolerance in plants. These MYB TFs include MdMYB23, MdMYBPA1, MdMYB1, and MdMYB308L. MdMYB23 directly binds to the promoter of *MdANR*, a crucial regulator of proanthocyanidin biosynthesis and accumulation, as well as ROS scavenging [69]. Furthermore, it plays a role in enhancing the cold tolerance of apples through the CBF-dependent pathway. This is achieved by binding to the promoters of *MdCBF1* and *MdCBF2* and stimulating their expression [69]. MdMYBPA1 is another positive regulator in response to cold stress conditions by facilitating anthocyanin accumulation. It interacts with MdMRLK2, which is a FERONIA receptor-like kinase, to promote the binding of MdMYBPA1 to the promoters of *MdANS* and *MdUFGT*, which are key genes involved in anthocyanin biosynthesis. This interaction leads to the activation of their expressions for the accumulation of anthocyanin [98]. Moreover, MdBBX20 interacts with MdHY5 and enhances the promoter activity of *MdMYB1*, which mediates the accumulation of anthocyanin in response to low temperature [24]. Finally, MdMYB308L enhances anthocyanin accumulation and cold tolerance in apple. It interacts with MdbHLH33 to increase the binding of MdbHLH33 to *MdCBF2* and *MdDFR* (an anthocyanin biosynthesis-related gene). This interaction leads to anthocyanin accumulation and thereby positively regulating cold tolerance in apple [73] (Table 3).

In addition to normal transcription regulation, complex feedback regulation is a crucial method for apples to respond to cold stress conditions. One such example is the regulation of *MdCBF3* promoter by the transcription factor MdMYB108L, which functions as a positive modulator in response to cold stress. Upon exposure to cold, MdHY5 activates the expression of *MdMYB108L*, which in turn downregulates the transcription of *MdHY5* [72]. This feedback loop enables precise regulation of gene expression and enhances the ability of apples to respond to cold stress in a more efficient and effective manner.

MdMYB94 has been found to bind to the promoter of *MdGH3.6*, an enzyme that participates in the conjugation of the plant hormone indole-3-acetic acid (IAA), thereby inhibiting its expression and promoting the biosynthesis of leaf cuticular wax, which ultimately confers drought tolerance in apple tree [45]. This finding is significant because MdGH3.6 is known to negatively regulate apple drought tolerance by mediating root development, leaf cuticular wax production, and secondary metabolites. Interestingly, when *MdGH3.6* RNAi plants are used as rootstock, the grafted plants exhibited enhanced growth vigor, improved water use efficiency, and even induced flowering and fruiting, highlighting the potential of this approach in breeding stress-resistant rootstocks [45, 99].

#### bHLH regulon

bHLH transcription factors are identified based on the bHLH domain, which contain 13–17 conserved amino acids and function by binding to the specific E-box (CANNTG) motif in the promoters of its target genes [100, 101]. In apple genome, 188 bHLH genes have been classified into 18 subgroups based on phylogenetic analysis [100]. Recent studies indicate that certain bHLHs may contribute to the apple’s ability to respond to abiotic stress [100, 101]. For example, MdbHLH130 and MdbHLHm1 have been identified as drought-positive factors. Under drought conditions, MdbHLH130 improves the tolerance of transgenic tobacco to soil water deficits by increasing stomatal sensitivity to ABA and upregulating the expression levels of ROS-scavenging and stress-responsive genes [27]. Similarly, overexpression of MdbHLHm1 leads to increased tolerance to salt stress and improved drought resistance in transgenic apple calli and Arabidopsis [25]. The bHLHm1 transcription factor MdSAT1 also facilitates transgenic Arabidopsis and apple calli drought tolerance by modulating the expression of stress-related genes (*ZEP*, *NCED9*, *AAO3*, *P450*, *RD16*, *RD29A*, *KIN2*, and *DREB2*) and decreasing their MDA contents, relative electrolyte leakage, and H_2_O_2_ content. Additionally, overexpressing *MdbHLHm1* confers salt stress tolerance in transgenic plants [25]. These findings indicate the multiple roles of MdbHLHm1 in abiotic stress responses, as it is also involved in cold stress response [25]. In addition to MdSAT1, MdCIbHLH1, an ICE-like protein, also functions in enhancing chilling tolerance in transgenic tobacco *via* the CBF-dependent pathway [70]. Specifically, MdCIbHLH1 associates with MdERF1B and mediates the binding of MdERF1B to the promoters of *MdERF3* and *MdCBF1*. This, in turn, promotes the ethylene biosynthesis and enhances the cold stress tolerance of apple trees [26]. These findings suggest that bHLH TFs have significant functions in controlling the response of apple to abiotic stress. By modulating the expression of genes related to stress and promoting ethylene biosynthesis, these factors can improve the ability of apple trees to withstand different environmental stresses. More research is required to fully understand the mechanisms underlying the functions of bHLH TFs in apple’s stress response. This will aid in devising effective strategies for enhancing apple’s stress tolerance.

#### NAC regulon

As well as MYB, bHLH, and BBX, the NAC transcription factor also plays a crucial role in apple’s response to cold stress through the CBF-dependent pathway. The NAC TF MdNAC029 has been found playing a negative role in apple cold tolerance. Overexpression of *MdNAC029* in Arabidopsis and apple calli leads to a cold-sensitive phenotype by repressing the expression of cold-responsive genes, including *MdCBF* genes (*MdCBF1*, *MdCBF2*, *MdCBF3*, *MdCBF4*, and *MdCBF5*), as well as their downstream target genes (*MdKIN1*, *MdRD29A*, and *MdCOR47*) [19]. Furthermore, NACs also participate in apple’s response to drought stress. Overexpression of *MdNAC1* or *MdNAC143* enhances apple’s drought tolerance by increasing the photosynthetic rate and activity of ROS-scavenging enzymes [18, 48]. Besides, another key NAC transcription factor, MdSND1 functions as an important role in apple’s response to salt and osmotic stress. It binds to the promoter of *MdMYB46/83* to mediate the lignin biosynthesis, which in turn improves the apple’s salt and osmotic stress tolerance [77].

#### WRKY regulon

In apple genome, there are 127 WRKY transcription factors that can be clustered into four subgroups based on their WRKY domains and the zinc-finger motif [29]. Among these, the group IIa WRKY genes (such as MdWRKY30) play as positive modulators in response to salt and osmotic stress conditions by activating stress-responsive genes [28]. Additionally, MdWRKY100 is another member of the WRKY family that participates in apple salt stress tolerance, and its promoter can be activated by MdSPL13. Overexpressing both *MdWRKY100* and *MdSPL13* in transgenic apple plants has been shown to enhance salt tolerance by increasing relative water and chlorophyll contents, while decreasing MDA and H_2_O_2_ levels [83]. Interestingly, the miR156-SPL module has also been found to regulate the expression of *MdWRKY100* under salt stress conditions [83]. Moreover, MxWRKY55, isolated from *Malus xiaojinensis* which is tolerant to abiotic stresses, has been found as a positive modulator of apple salt stress tolerance. Overexpression of *MxWRKY55* in Arabidopsis facilitates plant salt tolerance by activating antioxidant enzymes, including SOD, POD, and CAT [84]. Similar phenotypes have been found in the *MxWRKY64* and *MxWRKY53* overexpression Arabidopsis transgenic plants in response to salt stress [85, 86]. In addition to *M. xiaojinensis*, *M. baccata* is also highly tolerant to both cold temperatures and disease infections [65]. A series of WRKY TFs isolated from *M. baccata*, including MbWRKY1 [49], MbWRKY2 [51], MbWRKY3 [50], MbWRKY4 [87], and MbWRKY5 [46] have been identified as participating in responses to the drought and salt stress. Ectopic overexpression of these genes in tobacco plants enhances their ability to scavenge ROS, resulting in increased tolerance to drought and salt stress [46, 49–51, 87]. Furthermore, under drought stress, MdWRKY17 has been found to be a positive regulator of apple chlorophyll metabolism. It directly binds to the promoter of *MdSUFB*, a crucial component of the sulfur mobilization (SUF) system responsible for assembling Fe-S clusters. This activation leads to an increase in *MdSUFB* expression, which inhibits chlorophyll degradation and stabilizes electron transport during photosynthesis [57]. As a result, overexpression of *MdWRKY17* can lead to enhanced chlorophyll stability and activated photosynthesis, which contributes to the drought tolerance of the plants [57].

### Post-transcriptional regulation

**Figure 2:**
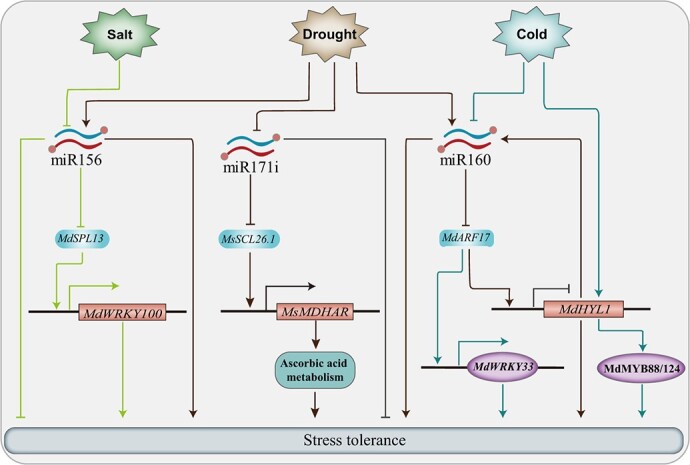
Apple miRNAs are involved in responding to cold, drought, and salt stress.

MicroRNAs (miRNAs) are a class of short non-coding RNAs that regulate the expression of target genes at the post-transcription level during stress responses, including in apple [83, 102–104] (Fig. 2). The identification of miRNAs in apple has been facilitated by deep small RNA-seq analysis, including 23 conserved, 10 less-conserved, and 42 apple-specific miRNAs or families with unique expression patterns [105].

Of these miRNAs, miR156 has been found to have the highest read abundance and is expected to target nine members of the SPL (squamosa promoter-binding-like) protein family. Further study using RLM-5′RACE confirms that *MdSPL13* (a negative regulator under salt stress) is the target gene of miR156 [83]. When apple plants are exposed to salt stress, the downregulation of miR156 leads to an enhanced expression of *MdSPL13*, which subsequently binds to the GTAC sites in the promoter of *MdWRKY100*, thus activating its expression to confer tolerance to salt stress [83]. In a study using F1 progeny and Illumina sequencing to identify key drought responsive microRNAs using drought-tolerant and -sensitive genotypes, overexpression of miR156 and miRn-249 significantly improve the tolerance of transgenic apple calli to drought stress stimulated by PEG [106]. Additionally, the abundance of miR156 is regulated by a key protein involved in miRNA biogenesis – MdSE (SERRATE) – which plays a negative role in apple drought tolerance [43].

Like miRNA156, the miRNA171li sequence is conserved among plant species and plays a crucial role in regulating drought stress tolerance in apple. Specifically, miR171i directly targets the *MsSCL26.1* (SCARECROW-LIKE PROTEINS26.1) and participates in drought stress tolerance by regulating ascorbic acid metabolism. MsSCL26.1 can bind to the promoter of *MsMDHAR* to activate its expression and facilitate ROS scavenging. Overexpression of *MsSCL26.1* confers the drought tolerance of the plants. Furthermore, knockout of miR171i by CRISPR/Cas9-mediated genome editing reduces the degradation of *MsSCL26.1*, which in turn enhances the drought tolerance of apple [104].

Another miRNA involved in apple drought tolerance is miRNA160. This particular miRNA targets the transcription factor MdARF17, which is a negative modulator of drought tolerance by suppressing the transcript level of *MdHYL1* (HYPONASTIC LEAVES1), a gene involved in drought-responsive miRNA biogenesis. As such, MdARF17 negatively regulates the biogenesis of drought-responsive miRNAs, including miR160. Therefore, the positive feedback regulatory loop formed by the miR160-MdARF17-MdHYL1 module helps to modulate apple drought stress. Interestingly, research has shown that miR160 can enhance the drought tolerance of apple trees by improving root development. This is possible because miR160 can move from scion to rootstock [12]. Furthermore, the Mdm-miR160–MdARF17–MdWRKY33 module mediates apple freezing tolerance by regulating reactive oxygen species scavenging [13]. Unlike its role in response to drought stress, however, miR160 actually serves as a negative regulator in response to cold stress conditions. When exposed to cold environments, an increase of miR160 and miR156 are observed in *MdHYL1* RNAi transgenic apple plants, which are cold sensitive. On the other hand, research has identified another microRNA called miR172 as being a positive regulator of cold tolerance [107]. Additionally, it seems that MdMYB88 also plays a part in regulating microRNA biogenesis through its interaction with MdHYL1 [108].

Currently, research has found that miRNAs function powerful roles in plant growth, development, metabolism, and stress response. However, there is still a lack of deep studies on miRNAs in apple. Only a few studies have investigated the association between miRNA expression and apple abiotic stress response, and their molecular mechanism remains unclear. Although the functions of miRNAs are conserved among different plant species, the target genes of miRNAs are more variable in apples due to the complexity of the genome. Therefore, identifying key miRNAs in apples and revealing their roles in regulating abiotic stress at molecular level will be important for future research.

### Post-translational regulation

Protein post-translational modifications, including phosphorylation, methylation, acetylation, SUMOylation, ubiquitination, and glycosylation, involve a variety of cellular processes that mediate the addition of chemical groups following protein translation, often resulting in changes in protein functions [109–111]. These protein post-translational modifications provide apple trees with a faster and more effective defense against abiotic stresses.

#### Phosphorylation

Phosphorylation is a widely studied post-translational modification that has been observed in apple. For instance, low temperatures can induce the expression and phosphorylation of dehydrins such as MdDHN2 and MdDHN4113 [112]. Additionally, the glucose sensor hexokinase1 (MdHXK1) interacts with and phosphorylates MdNHX1 (a vacuolar Na^+^/H^+^ antiporter) at its Ser-275 residue to improve its stability, thereby enhancing its tonoplast Na^+^/H^+^ transport activity. This ultimately improves salt tolerance in apple [113]. Another example is the novel AtSOS2-LIKE protein kinase, MdSOS2L1, which can phosphorylate MdVHA-B1 (a V-ATPase subunit) at Ser-396 to enhance V-ATPase activity and promote malate accumulation in apple for improved salt stress tolerance [114]. In terms of sucrose transporters, MdSUT2.2A plays various roles in abiotic stress responses in apple through phosphorylation at different serine sites. Under salt stress conditions, the protein kinase MdCIPK13 mediates the phosphorylation of MdSUT2.2A at Ser254 to improve its stability and activity for enhanced salt tolerance of apple calli [115]. On the other hand, drought-induced phosphorylation of MdSUT2.2A at Ser381 mediated by MdCIPK22 improves drought tolerance by promoting sugar accumulation [116]. Finally, during drought conditions, the drought-induced gene MdWRKY17 is phosphorylated on Ser66 by the cascade consisting of MdMEK2–MdMPK6 [57]. The activation further strengthens chlorophyll stability and photosynthesis via activating the MdWRKY17–MdSUFB pathway leading to increased drought resistance [57] (Table 4).

**Table 4:** Post-translational regulation of apple in response to abiotic stresses

Protein modifications	Substrate	Modification site	Interacting proteins	Functional remarks	Abiotic stresses
Phosphorylation	MdSUT2.2	Ser254	MdCIPK13	Sucrose transport activity	Salt [115]
	MdSUT2.2	Ser381	MdCIPK22	Sugar accumulation	Drought [116]
	MdVHA-B1	Ser396	MdSOS2L1	V-ATPase activity, malate accumulation	Salt [114]
	MdNHX1	Ser275	MdHXK1	Na^+^/H^+^ transport activity	Salt [113, 127]
	MdWRKY17	Ser66	MdMPK6	Chlorophyll stability	Drought [57]
SUMOylation	MdMYB1 MdDREB2A	K172	MdSIZ1 MdSCE1	Anthocyanin biosynthesis Protein stability	Cold [122] Drought [61]
Ubiquitination	MdBBX37		MdMIEL1	CBFs expression	Cold [91]
	MdNAC143		MdBT2		Drought [48]
	MdBBX7		MdMIEL1	MdGLK1/ERF1/ERD15 expression	Drought [23]

#### Ubiquitination

Besides phosphorylation, ubiquitination is another kind of post-translational modification widely involved in apple stress response. It is the process by which a protein is covalently modified by the addition of a small ubiquitin molecule. The labeling of proteins with ubiquitin is accomplished by the action of three enzymes: E1 (activating enzyme), E2 (conjugating enzyme), and E3 (ubiquitin ligase). The ubiquitinated protein is subsequently degraded through the 26S proteosome pathway [111, 117]. Among the three enzymes, E3 is responsible for the specificity of ubiquitination processing. MdMIEL1 is an E3 ligase that functions in the stress response of apples. It interacts with MdBBX37 to facilitate its degradation, which ultimately leads to reduced apple cold tolerance [91]. Furthermore, under drought stress conditions, MdMIEL1 mediates the ubiquitination of MdBBX7 [23]. Another negative regulator in apple anthocyanin biosynthesis and leaf senescence is the scaffold protein MdBT2. This protein has ubiquitination activity and forms complexes with CUL3 and RBX1 [118–121]. The interaction between MdBT2 and transcription factor MdNAC143 negatively regulates drought tolerance in apples under drought stress conditions [48] (Table 4).

#### SUMOylation

Unlike ubiquitination, which is a well-known protein modification that induces proteasomal degradation, SUMOylation (small ubiquitin-like modifiers) has been found to enhance the stability of substrate proteins in most plant studies [122, 123]. Although both ubiquitination and SUMOylation involve covalent attachment to target proteins, they differ significantly in their functions and mechanisms. Specifically, SUMOylation modifies lysine residues on target proteins by attaching SUMO proteins, leading to changes in activity, subcellular localization, and interactions [122, 124]. The SUMOylation of target proteins is mediated by both the SUMO E2-conjugating enzyme (SCE1) and the SUMO E3 ligase [61, 125]. In apple, a SUMO E3 ligase, MdSIZ1, facilitates the regulation of lateral root formation through the SUMOylation of MdARF8 [126]. Additionally, it targets MdbHLH104 to mediate the SUMOylation of MdbHLH104 to regulate iron homeostasis and plasma membrane H^+^-ATPase activity [127]. A recent study has shown that SUMOylation of MdMYB1 by MdSIZ1 enhances the stability of MdMYB1 and promotes anthocyanin accumulation under low temperature conditions [122]. Additionally, the fine-tuning regulation role of SUMOylation in apple’s response to drought stress is revealed. Both the increased and decreased levels of SUMOylation have led to increased drought tolerance in apples under drought stress conditions. Overexpression of *MdSUMO2A* results in plants with a well-developed root system, more vigorous growth, higher photosynthetic capacity, and increased hydraulic conductivity – acting as water spenders. Conversely, the RNAi plants with reduced levels of *MdSUMO2*s act as water savers with smaller leaves, thicker leaves, lower stomatal conductance but higher water use efficiency [61]. Further study identified MdDREB2A as a substrate of MdSUMO2s; however, unlike previous studies showing that SUMOylation facilitates protein stability, SUMOylated MdDREB2A is recognized by RING-finger protein (MdRNF4, also known as SUMO-targeted ubiquitin E3 ligase) and degraded *via* the 26S proteosome pathway during drought stress [61].

### Epigenetic modification regulation

Chromatin structure usually affects the expression of the genome. And the chromatin structure is governed by the process, which is frequently connected with epigenetic modification regulation, including DNA and RNA methylation and histone modifications. These epigenetic regulations also play critical roles in stress responses of apple.

#### DNA methylation

DNA methylation and histone methylation/acetylation are heritable information that modulates gene expression without changing the DNA sequence [128]. In apple genome, single-base methylome analysis shows the potential linkages between DNA methylation and gene expression during drought stress [129]. A comparison of the methylome and transcriptome between a drought-tolerant and a drought-sensitive apple reveals that the genes with unmethylated promoters exhibit higher expression levels compared to those with methylated promoters [129]. In *M. prunifolia*, a species tolerant to drought, heat, and cold stresses [130], lower methylation levels in MpDREB2A have been associated with its higher expression level contributing to its tolerance against droughts [54]. Furthermore, genome-wide DNA methylation and RNA-seq analyses provide insights into grafting-mediated stress tolerance in *M. prunifolia* by identifying differentially methylated regions in promoters of genes involved in environmental adaptation, flowering, and ABA biosynthesis induced by stress stimulation; these stress-related genes are contributed by *M. sieversii* and *M. baccata* species [130]. Genomic bisulfite sequencing reveals that low-temperature exposure leads to demethylation of anthocyanin biosynthetic gene promoters resulting in increased anthocyanin accumulation due to elevated gene expression levels [131]. The genomic DNA methylation level also changes during chilling, which is necessary for apple dormancy in the winter [132].

#### m6A

In recent years, increasing evidence has demonstrated the involvement of m6A (N6-methyladenosine) in various aspects of mRNA metabolism such as RNA stability, translation efficiency, nuclear-cytoplasmic export, and pre-mRNA splicing [133]. In apple genome, the transcriptome-wide m^6^A methylome profiling shows that m^6^A is predominantly enriched in the coding sequence and untranslated region upon drought stress [134]. MdMTA is a m6A writer that enhances mRNA stability and translation efficiency of genes responsible for lignin deposition and oxidative stress, thereby positively regulating drought stress tolerance [134]. Furthermore, the m6A reader known as MhYTP2 plays a crucial role in apple powdery mildew stress. It achieves this by increasing both the mRNA stability of MdMLO19 and the translation efficiency of genes related to antioxidants [135].

To date, there have been very few studies on the molecular mechanisms of epigenetic modification regulation in apple’s response to abiotic stress. Specifically, the involvement of histone variants and post-translational modifications in plant stress response has been reported in Arabidopsis but not yet in apple. Therefore, gaining an understanding of how epigenetic modifications such as DNA and RNA methylation, histone variants, and post-translational modifications participate in regulating apple’s response to abiotic stress would be valuable for future molecular breeding efforts.

### Molecular approaches to facilitate apple tolerance to abiotic stress

Traditional breeding in woody species is a slow process for improving plants, because of their long juvenility and the high level of heterozygosity caused by the self-incompatibility system. Genetic engineering can provide valuable references for efficient breeding [136]. The most recent discoveries in the field of molecular biology have led to the development of ‘new breeding technology’ (NBTs) or ‘technology of assisted evolution’ (TEAs) that include cisgenesis and genome editing. Compared with conventional breeding methods, TEAs are greatly reducing the time required to obtain new varieties, allowing for targeted intervention in genes of interest without altering all the other traits appreciated in each cultivar by the market. The development and use of NBTs has been greatly facilitated by the availability of an ever-increasing number of apple genome assemblies since the publication of the first sequence of ‘Golden Delicious’ [137]. The publication of the assembly of the multi-stress resistant cultivar ‘Hanfu’ will provide an invaluable instrument for studying the genetics of abiotic stress resistance in apple [138].

#### Genome editing

Among the NBTs, the CRISPR/Cas9-FLP/FRT-based gene editing system provides a new direction for modern apple molecular breeding, which produces T-DNA free CRISPRed apple trees [10, 139]. In 2016, the first successful endogenous DNA-free genetically edited apple was reported using CRISPR/Cas9 ribonucleoproteins. Specifically, an apple phytoene desaturase (PDS) gene is precisely modified with the application of guide RNAs (gRNAs) and Cas9 ribonucleoproteins. Among all the transformants, about 31.8% of the apple plantlets demonstrate a clear albino phenotype, indicating that genome editing methodologies can be applied to apple breeding programmes [140]. In addition to PDS gene modification, three susceptibility genes to fire blight (*DIPM-1*, *DIPM-2*, and *DIPM-4*) are site-directed mutated in apple protoplasts [9], This highlights the potential for generating exogenous DNA-free genome edited apples with high resistance to fire blight. Three years later, the editing efficiency of *MdPDS* reachs over 85% with the improvement of the CRISPR/Cas9 system, including promoters (gRNA and Cas9 promoters) and gRNA sequencings [141]. At the same time, Terminal Flower 1 (TFL1) gene is successfully edited using ‘Gala’ transgenic lines expressing the CRISPR-TFL1.1 construct which results in flowering between one to six months after micropropagation [141]. Moreover, the CRISPR/Cas9 technology is also used successfully to introduce the albino phenotype in *M. sieversii* in 2021 [142]. Finally, a high-efficiency gene editing system based on CRISPR/Cas9-FLP/FRT has been successfully employed to excise the T-DNA containing the expression cassettes for CRISPR/Cas9, the marker gene, and FLP itself. This has resulted in the production of an edited apple plant that carries only a minimal amount of exogenous DNA [10]. Nowadays, studies on apple genome editing mediated by the CRISPR/Cas9 system are mainly focused on leaf color, flowering, and biotic stress tolerance. However, further investigation is necessary to utilize this system as a crucial molecular breeding technique for enhancing abiotic stress tolerance in apples.

#### Molecular markers

QTL (quantitative trait locus)-based functional markers play a significant role in apple molecular breeding through marker-assisted selection (MAS). However, current studies primarily concentrate on identifying functional DNA markers related to fruit traits and biotic stress resistance, while only a few studies on abiotic stresses have been reported.

Two functional markers, SNP182G and SNP11G/SNP761A, have been identified in progenies of the cross between ‘Baleng Crab’ (*Malus robusta* Rehd.) and ‘M9’ (*Malus pumila* Mill.), which confer salt stress tolerance [143]. The former is located in the *MdRGLG3* gene on linkage group 16 (LG16) and causes a leucine to arginine substitution at the vWFA-domain, thereby improving apple tree tolerance to salt, alkali, and salt-alkali stress. The latter marker is found in the *MdKCAB* gene on LG16 as well and affects the Kv_beta domain that cooperates with linked allelic variation SNP11; it also contributes to tolerance in a similar manner as SNP182G. Recently, natural variation among *Malus* accessions has been discovered to be responsible for apple drought stress tolerance. The insertion of a miniature inverted-repeat transposable element (MITE) in the promoter of *MdRFNR1–1* is hypermethylated and recognized by transcriptional anti-silencing factors MdSUVH1 and MdSUVH3. These factors facilitate the recruitment of DNAJ domain-containing proteins, including MdDNAJ1, MdDNAJ2, and MdDNAJ5, which in turn activate the expression of *MdRFNR1–1* under conditions of drought-induced stress [11]. MdRFNR1 exerts a beneficial effect on drought response by regulating the redox system, which involves upregulation of NADP^+^ accumulation, catalase and peroxidase activities, and downregulation of NADPH levels [11]. *Malus* accessions harboring this MITE insertion within the *RFNR1* promoter exhibit elevated levels of POD and CAT activities, as well as reduced MDA content, compared to those lacking this insertion under drought conditions. This suggests that this natural variation could serve as an efficient molecular marker for breeding apple trees with improved drought resistance.

## Concluding remarks

In this review, we have summarized the molecular regulation mechanisms of apple under abiotic stress conditions, including drought, extreme temperature, and salt stresses. Identifying the factors that determine abiotic stress tolerance is crucial for enhancing apple performance under such conditions. This can be achieved through the use of NBTs, including genetic engineering, as well as traditional breeding techniques using MAS. Although significant progress has been made in identifying molecular regulatory mechanisms of apple in response to abiotic stress over the last few decades, including transcriptional mediation and post-transcriptional and post-translational modulation, key regulators of abiotic stress responses in apple and their underlying regulatory mechanisms remain unclear. The implementation of CRISPR/cas9 for precise DNA editing in apple and improving breeding efficiency remains challenging due to a lack of an effective transformation system and high-quality heterozygous genome. Studies combining bioinformatics, molecular biology, and physiology are particularly useful for improving apple stress tolerance and breeding efficiency.
